# The Latest Achievements of Liquid Membranes for Rare Earth Elements Recovery from Aqueous Solutions—A Mini Review

**DOI:** 10.3390/membranes13100839

**Published:** 2023-10-21

**Authors:** Małgorzata A. Kaczorowska

**Affiliations:** Faculty of Chemical Technology and Engineering, Bydgoszcz University of Science and Technology, 3 Seminaryjna Street, PL 85326 Bydgoszcz, Poland; malgorzata.kaczorowska@pbs.edu.pl

**Keywords:** rare earth elements, separation, supported liquid membrane, emulsion liquid membrane, polymer inclusion membrane

## Abstract

The systematic increase in the use of rare earth elements (REEs) in various technologically advanced products around the world (e.g., in electronic devices), the growing amount of waste generated by the use of high-tech materials, and the limited resources of naturally occurring REE ores resulted in an intensive search for effective and environmentally safe methods for recovering these elements. Among these methods, techniques based on the application of various types of liquid membranes (LMs) play an important role, primarily due to their high efficiency, the simplicity of membrane formation and use, the utilization of only small amounts of environmentally hazardous reagents, and the possibility of simultaneous extraction and back-extraction and reusing the membranes after regeneration. However, because both primary and secondary sources (e.g., waste) of REEs are usually complex and contain a wide variety of components, and the selectivity and efficiency of LMs depend on many factors (e.g., the composition and form of the membrane, nature of the recovered ions, composition of the feed and stripping phases, etc.), new membranes are being developed that are “tailored” to the properties of the recovered rare earth elements and to the character of the solution in which they occur. This review describes the latest achievements (since 2019) related to the recovery of a range of REEs with the use of various liquid membranes (supported liquid membranes (SLMs), emulsion liquid membranes (ELMs), and polymer inclusion membranes (PIMs)), with particular emphasis on methods that fall within the trend of eco-friendly solutions.

## 1. Introduction

The group of rare earth elements (REEs) includes 15 elements classified as lanthanides, as well as scandium and yttrium, which are characterized by similar chemical properties and are often found in the same minerals as the lanthanides [1]. Due to their properties, REEs are used in a variety of industries, including intensively developing, so-called “green technologies”, e.g., related to the production of equipment for wind energy generation or batteries for electric cars. The applicability of these elements is very wide, as they play an important role in various electronic sectors, in the optical industry, in the production of high-performance magnets, in agriculture, in the petroleum industry, and in military-related industries [2,3]. The systematic increase in the consumption of REEs in various fields and the limited supplies of ores containing these elements mean that they are recognized as critical raw materials in many countries (e.g., in the European Union). Moreover, due to the importance of rare earth elements in modern industry, an increase in the demand for these materials may grow, even by around 8% annually [4,5]. Attempts to ensure permanent and stable access to REEs have resulted in an increased interest in technologies that allow the acquisition of these critical raw materials from secondary sources [6,7]. Significant secondary sources include but are not limited to contaminated soil, combustion ashes, mine sediments and tailings, used magnets, and various types of electronic waste [2,8]. Recovery of REEs from these sources is also an important activity contributing to the development of the circular economy, which has a significant and beneficial impact on the natural environment [9,10]. However, the content of REEs in different secondary sources varies, as it can range from trace amounts to several dozen percent (e.g., waste NdFeB magnets contain about 30 wt%) [5]. Effective recovery of rare earth elements from secondary sources requires the development of methods adapted to the nature of the source (including the amount and type of REEs contained), which are also safe for the environment and economical, and enable the recovery process to be carried out with satisfactory efficiency. For example, for the recovery of REEs from rare earth mine wastewater, chemical precipitation, ion exchange, solvent extraction, membrane separation, and adsorption have been used, among others, and recently research is also being carried out on the applicability of synthesized bio-nanoparticles (e.g., derived from Bacillus cereus) [11]. Particular attention has been paid in recent years to environmentally friendly methods that allow the efficient recovery of REEs from waste electrical and electronic equipment (WEEE), the amount of which is systematically growing due to technological and economic development [12]. In the case of recovery of REEs from WEEE, both physical processes (e.g., based on the use of magnetic properties) and chemical processes (e.g., pyrometallurgical, hydrometallurgical, bio-metallurgical, or electrochemical) are used. However, recovering REEs from e-waste is not easy due to the complexity of the material and the content of various substances, including toxic heavy metals, that are dangerous to the environment. Therefore, for example, in some hydrometallurgical processes applied, shredded e-waste, free of undesirable components (e.g., glass, plastic), is leached (e.g., with strong acids), and then metal ions are selectively separated from the obtained solution using various techniques [13,14]. Because REEs have similar properties (e.g., they can form stable trivalent ions of similar size in solutions), their separation is usually neither easy nor cheap, and many of the developed advanced separation techniques lead to the generation of large amounts of hazardous waste (e.g., radioactive) [15].

In recent years, various membrane techniques have been used to remove REEs from different materials (including rare earth ores, waste, and wastewater) because they usually allow not only the selective separation of metal ions but are also reliable, energy-saving, and easy to scale [16]. In general, membranes are barriers separating two different phases, which allows for the selective separation of the mixture of chemical compounds, bypassing some substances and retaining others. They can be categorized based on criteria such as, inter alia, origin (e.g., synthetic, natural), morphology (e.g., porous, non-porous), or driving force (e.g., microfiltration, reverse osmosis) [16,17,18]. One of the types of membranes successfully used to remove or recover different metal ions from various solutions (sources) is a liquid membrane (LM), which enables the solvent extraction and stripping processes in a single step. Typically, LM-based methods have significant advantages over solvent extraction, such as higher separation efficiency and lower consumption of chemical reagents, which also makes them more environmentally friendly. Usually, in LM techniques, a three-phase system is created in which there is an organic phase (immiscible or semi-permeable, held stationary or circulating in the system) and feed and stripping mobile aqueous phases. LMs consist of a solvent, an active carrier that transports certain components, e.g., REE ions, and some auxiliaries. Due to differences in form, LMs can be divided into three major groups: bulk (BLM), supported (SLM), and emulsion (ELM) membranes. Differences in form between various LMs are quite significant, e.g., in the case of SLM, a porous material (polymer) is impregnated with the organic liquid membrane, while in ELM, the membrane solvent is emulsified. However, because of certain limitations associated with the use of LMs (e.g., SLM and ELM often exhibit low stability during application), the possibility of membrane modifications that would enable more efficient membrane processes, also on a larger scale, have been systematically investigated [19,20]. A type of liquid membrane whose composition and thickness can be easily modified is the polymer inclusion membrane (PIM), which, in addition to the polymer that forms the matrix of the membrane and the ions binding carriers, also usually contains a plasticizer that gives the membrane adequate plasticity. Research related to LM modifications is carried out both in terms of changing the composition of membranes (e.g., replacing one compound with another, which refers to carriers, solvents, polymers, and plasticizers), using a combination of different chemical compounds instead of single ones (e.g., utilization of two different carriers), and changing the amounts of components and the conditions of membrane processes (e.g., composition of the feeding and stripping phases, process duration, temperature, pH, etc.). The type of membrane used and its composition as well as the conditions of the membrane process should be properly matched to the properties of recovered metal ions and the matrices in which they occur, which is often not easy due to their complexity. What is more, the membrane-based method that is optimal for recovering one type of metal ion from a specific source may not work for other metal ions, and even a slight change in the conditions of the process can significantly affect its effectiveness [21,22].

This article reviews the latest achievements (primarily from 2019) in the use of various types of liquid membranes for the recovery of rare earth elements from different aqueous solutions and discusses the essential advantages and disadvantages of the developed techniques.

## 2. Recovery of REEs with the Use of Supported Liquid Membranes

In general, in supported liquid membrane extraction, the aqueous feed and stripping phases are separated with a thin membrane in which the organic phase is immobilized, and organic carriers located in the micropores of the support enable the transport of metal species. The separation of metal ions using SLM can be considered a simultaneous three-stage process consisting of extraction of ions from the feed phase to the SLM, diffusion through the SLM, and stripping process to the receiving phase. Therein, selective transport is related to different ion permeabilities, which are connected, inter alia, to the driving force and the thickness of SLMs [23,24]. However, it should be noted that supported liquid membranes can be used in the form of flat sheet membranes (FSHSLMs) or hollow fiber membranes (HFSLMs), and the form of an SLM may also influence the membrane process [23]. In the case of HFSLMs, the membrane phase is held by capillary forces in the pores of microporous hollow fibers forming special modules (thin fibers placed along the length of the shell) through which the feed phase is pumped, whereas the stripping phase is forced out through the sides of the shell [25]. It has been reported that HFSLMs have a higher surface area than FSHSLMs and provide more rapid transport, with feed and stripping phases more easily recoverable, and the entire feed and receiving phases are not in contact with the membrane [26]. The differences between the two types of SLMs also relate to the materials used for their formulation, e.g., for support of the carrier phase in FSHSLMs, among others, porous polytetrafluoroethylene (PTFE) or polyvinylidenedifluoride (PVDF) [27,28] can be used, in the case of HFSLMs, polypropylene fibers are often utilized [29].

One of the key factors influencing the SLM processes is the properties of the carriers used, and, therefore, many studies have been conducted on the possibility of using various chemical compounds for this purpose. Currently, while well-known carriers are used (introduced modifications involve the parameters of membrane processes), new substances are also sought that could prove to be more efficient carriers. Due to the differences in properties, carriers are most often divided into groups of basic, acidic and chelating, macrocyclic and macromolecular, and neutral and solvating compounds [30]. Among well-known acidic carriers, di-(2-ethylhexyl)phosphoric acid (D_2_EHPA) is widely used in various types of liquid membranes intended to remove metal ions [22]. The advantages of D_2_EHPA are, among others, high selectivity toward REEs and miscibility with most of the common diluents. Recently, D_2_EHPA has been used in several methods intended for the recovery of different REEs from various sources. For example, Ni’am et al. [31] used a hollow fiber-supported liquid membrane module with hydrophobic microporous polypropylene hollow fiber support and D_2_EHPA (in Isopar-L) as the organic phase in the membrane for the recovery of neodymium ions from acidic leachate of waste permanent magnets and reported that the applied HFSLM enabled the recovery of 90.82% of the Nd in a short time process (35 min). They also examined the effectiveness of D_2_EHPA as an extractant in classical solvent extraction (SE) of neodymium ions and found that although SE efficiency was slightly higher (about 97% of recovered neodymium ions) than HFSLM separation, the membrane process was associated with the consumption of smaller amounts of chemical reagents (was more eco-friendly) and should be considered for industrial-scale development of REE recovery. Ni’am and co-workers [32], on the basis of results of performed SE and HFSLM experiments, reported that D_2_EHPA can also be successfully applied as an extractant/carrier for simultaneous recovery of rare earth elements, such as neodymium, dysprosium, and praseodymium ions from waste permanent magnet leach liquor. They found that in the case of the HFSLM process, the optimum transport rate was achieved at 90 min, and the transport of REE ions followed the order Nd > Pr > Dy. In addition, they demonstrated not only that HFSLM with D_2_EHPA is a promising and feasible technique for recovering REEs from waste permanent magnet leach solutions, but also that such membranes can be applied at a laboratory scale to recycle REEs from industrial waste. Recently, Mohdee et al. [33] showed that the use of HFSLM with D_2_EHPA, and with appropriately selected experimental conditions and carrier concentration, enabled high enrichment performance of Nd(III) ions and, under optimal conditions, extraction and stripping of neodymium ions reached 99.80% and 78.58%, respectively. Additionally, they applied density-functional theory (DFT) calculations for a detailed analysis of the reaction mechanism because the membrane process was controlled by mass transfer due to this chemical reaction. They reported that under optimal experimental conditions, the Nd(III)/D_2_EHPA molar ratio was 1:3, which was consistent with the obtained results of computational calculations, and that the coordinated covalent bonds between Nd(III) and D_2_EHPA were formed through six oxygen atoms. The analysis of the reaction mechanism is important because, although in general, during the reaction with di-(2-ethylhexyl)phosphoric acid, each trivalent REE ion is extracted in a complex with six molecules of D_2_EHPA arranged as dimers; other stoichiometric ratios were also reported in different experimental conditions [33,34]. One of the important limitations associated with the use of D_2_EHPA/HFSLM-based separation methods is the stability/durability of the membranes. As noted by Alemrajabi et al. [34], despite the many advantages of HFSLMs, this technology has not yet been implemented industrially mainly due to membrane instability, difficulty to scale-up, and relatively short lifetime of the membrane module. To find solutions that could potentially be used on an industrial scale, the abovementioned authors compared the recovery and separation of REEs from a synthetic feed solution (corresponding to apatite concentrate) with the application of a standard hollow fiber-supported liquid membrane, a renewal liquid membrane (HFRLM), and emulsion pertraction technology (EPT), using D_2_EHPA diluted in kerosene (10% *v*/*v*) as the organic membrane solution and 3 M HCl as stripping solution. In the HFRLM, the organic solution was soaked into polypropylene support and also uniformly dispersed into the stripping solution, whereas in EPT, the aqueous strip solution was dispersed in the organic solution. The results of that study indicated that the liquid membrane was more selective toward heavy REEs at a lower pH and a higher concentration of rare earth elements. The application of the HFRLM system enabled a higher transport rate than the use of the HFSLM, but the utilization of the HFSLM resulted in a higher selectivity toward individual metal ions. As the performance of the HFSLM deteriorated over time, as opposed to the relatively stable HFRLM and EPT, the authors concluded that the last two methods can be feasible options for the processing of REE leachates. The obtained results confirm that many factors influence the supported liquid membrane processes (initial composition and concentration of the feed phase, pH, form of the membrane, etc.) and the conditions of the recovery process should be investigated thoroughly when designing an SLM separation method for a specific multi-component REE solution. In many studies related to the recovery of a particular REE, scientists focus on the impact of process configuration optimization (choice of solvent, carrier, etc.) and/or the presence of other elements from the REE group on the selectivity and efficiency of the membrane process, but these parameters may also be significantly influenced by the presence of elements from outside the rare earth element group in the source/feed phase [32,35]. The presence of such “additional” elements may be of particular importance for the SLM recovery process of REEs from low-grade residuals such as mine wastes and combustion ash. For example, Middleton and Hsu-Kim [35] examined the recovery of neodymium and erbium ions from a model solution (with a composition corresponding to real leachates derived from coal ash and acid mine drainage, containing Fe^3+^, Fe^2+^, and Al^3+^ ions) using a flat sheet membrane composed of poly(vinylidene difluoride) support and D_2_EHPA carrier and reported that the absolute concentrations of iron and aluminum ions present in the feed phase controlled REE mass transfer, and that the permeability coefficients of Nd and Er ions were most sensitive to the concentration of Fe^3+^ (the threshold Fe^3+^ concentration that contributed to the reduction in Nd and Er permeability was more than 100 times lower than the concentrations required for Al^3+^ and Fe^2+^ to decrease the permeability of neodymium and erbium ions). They also reported that the pH gradient across the D_2_EHPA/FSHSLM and the relative cation affinity for the D_2_EHPA chelator were the major drivers for mass transfer. Interestingly, their results showed that the excess of Fe^3+^ ions in the feed phase did not cause any noticeable fouling of the membrane surface. The obtained results indicated which of the examined components of the feed solution can significantly influence the SLM-based recovery process of REEs from low-quality sources and, consequently, allow for better design of methods intended for this purpose. The development of REE recovery methods that can be successfully used in the case of leachates containing significant amounts of other substances (e.g., iron(III)) is important also because many crucial, secondary sources of rare earth elements are rich in such substances, e.g., acidic liquors obtained from waste permanent magnets [32].

Recently, SLMs containing other ion carriers have also been used for the recovery of various REEs. For example, Li et al. [24] used a flat sheet supported liquid membrane containing microporous polypropylene film and mono-(2-ethylhexyl) phosphoric acid (507P/EHEHPA) carrier for the efficient extraction and separation of Nd(III) and La(III) ions. They examined the influence of various factors on the FSHSLM process (i.e., Nd(III) and La(III) concentration in the feed phase, the concentration of EHEHPA in the membrane, acid solution concentration in the stripping phase) and reported that the extraction of La and Nd increases with the initial pH of the feed solution and carrier concentration. The development of an effective method for separating ions of both REEs is important because the recovery of neodymium ions from secondary sources, such as waste neodymium permanent magnets, usually requires prior Nd/La separation. Li et al. [36] also applied a flat sheet SLM with a series of alkylphosphorus compounds as extractants (507P/EHEHPA, or TBP—tri-*n*-butyl phosphate or 204P/D_2_EHPA), for Nd ion recovery from a La/Nd binary solution to compare the effectiveness of these compounds. Additionally, they examined different extractant–acid systems such as 204P-H_2_SO_4_, 507P-HCl, and TBP-HNO_3_, respectively. Their results indicated not only that 507P-HCl was the most efficient extractant for both the recovery of neodymium ions and Nd/La separation but also demonstrated the long-term stability of this system (during 6 days of the process). Another carrier used by Xu et al. [37] in SLM for the recovery of REE ions was *N*,*N*,*N*′,*N*′-tetraoctyl diglycolamide (TODGA), characterized by good extraction properties with relatively fast kinetics, high loading capacity for various lanthanides without formation of the third phase, and simple stripping of the extracted metal ions. Results of the performed experiments indicated that a flat sheet SLM with a TODGA carrier and PVDF support was highly selective for lanthanides and enabled effective transport of lanthanum(III), cerium(III), praseodymium(III), and neodymium(III) ions from the leaching solutions of phosphate ores (more than 95.0% of REEs was recovered using 0.10 M TODGA). Zarei et al. [38] examined the possibility of using the synergistic effect of organophosphorus extractants (mixtures of D_2_EHPA, TBP, and TOPO) to improve the recovery of lanthanum ions from aqueous solutions. They used an experimental design technique (central composite design approach) based on two-step optimization. First, the concentrations of the organophosphorus extractants, and then secondary parameters (i.e., composition of the feed and stripping phases, pH) were optimized to obtain the maximum permeability coefficient through the SLM. The said authors reported that the synergistic effect was noted when TBP was added to the D_2_EHPA extractant, and a non-synergistic or antagonistic behaviour was observed after the addition of TOPO to the D_2_EHPA extractant in the organic phase. In optimal experimental conditions, the extraction and stripping efficiencies were 56.89 and 49.88%, respectively. Davletshina et al. [39] compared membrane transport processes performed with the utilization of commercially available TOPO carrier or lipophilic phosphorylated betaine, hexyl[(*N*-methyl-*N*,*N*-dioctylammonio)methyl]phosphonate carrier. This compound, despite the known complexing and extracting properties of aminophosphabetaines, has not been used before in membrane processes intended for the recovery of REEs. Obtained results indicated that hexyl[(*N*-methyl-*N*,*N*-dioctylammonio)methyl]-phosphonate was more effective than the TOPO carrier for the recovery of triply charged rare earth metal ions, such as lanthanum, neodymium, and europium ions.

One of the trends in recent years in the field of SLM modification is the use of ionic liquids (ILs) as carriers because they usually extract metal ions well and are thermally stable, non-flammable, and characterized by good ionic conductivity and negligible vapor pressure. Ionic liquids, due to their properties, are often referred to as “green solvents” and are utilized in various conventional and advanced extraction techniques [40]. It has been reported that in the case of SLM, viscous ILs with low solubility in water can also improve membrane stability [41]. The formation of SLM membranes containing ILs is most often based on physical methods (such as impregnation, pressure-induced, and vacuum-induced methods), which lead to the impregnation of membrane pores (the basement of the membranes could be inorganic or organic with porous structures) with ionic liquids [42]. A relatively easy and economical procedure for the formation of SLMs, the availability of a wide range of ionic liquids (possibility of conducting efficient extraction in various experimental conditions), the environmental safety of the combination of membrane techniques with ILs acting as metal ion carriers, and the urgent need to develop effective methods for the recovery of REEs from aqueous solutions have resulted in a systematically growing interest in rare earth element separation methods based on the use of ILs/SLMs. Table 1 shows examples of various compounds, including ILs used during the last four years as carriers in SLMs intended for the removal of REEs from aqueous solutions.

It should be emphasized that ILs can be used in various configurations in SLMs, as alone (the only) carriers and as components of a mixture of two/several carriers (analysis of the possibility of synergistic action). Research conducted over the last years focused both on the comparison of the effectiveness of IL/SLM systems with supported liquid membranes containing traditional carriers and on the suitability of the same ionic liquid for the recovery of a variety of REE ions. For example, Asadollahzadeh et al. [43] compared the effectiveness of membrane processes intended for the recovery of cerium ions, based on the application of SLM with polytetrafluoroethylene support and imidazolium ionic liquid ([C_6_MIM][NTf_2_]) and organophosphorus (D_2_EHPA, TBT) extractants. Their results indicated that IL improved the extraction and increased the permeation coefficient through the SLM; however, the examined processes depended on parameters such as the acidity of the feed and stripping phases and the concentration of carriers (the maximum permeation coefficient was observed at 30%, 20%, and 10% *v*/*v* for D_2_EHPA, TBP, and [C_6_MIM][NTf_2_] concentrations, respectively).

Asadollahzadeh et al. [44] utilized the same IL in a study investigating the impact of the presence and absence of ionic liquid in the mixture of carriers used in an SLM designed for the extraction of praseodymium and neodymium ions from the leaching solution obtained from NdFeB magnets. They reported that the highest permeability coefficients were obtained with the synergistic system containing [C_6_MIM][NTf_2_], TOPO, and TPB extractants. Extraction efficiency in the case of using SLM with a mixture of TOPO and TBP carriers was about 74%, while the addition of IL to the mixture of extractants increased this value to over 90%. Additionally, the analysis of the stability of the examined SLMs demonstrated that a more stable system was provided with ionic liquid in the carrier phase. An increase in the efficiency of SLMs intended for the recovery of REE ions, as a result of the addition of ionic liquid [C_6_MIM][NTf_2_] to organophosphorus extractants was also observed in the case of processes performed for solutions containing gadolinium and yttrium ions [45,46]. However, if a mixture of carriers is used in SLM to increase the efficiency of REE ions recovery from aqueous solutions, determining the optimal conditions for the membrane process is often difficult. For example, Asadollahzadeh et al. [45] applied SLM with a mixture of ionic liquid, neutral, and acidic extractants ([C_6_MIM][NTf_2_], TBP, D_2_EHPA) and reported that although the carrier concentration is an important parameter influencing the membrane process, the concentrations of IL and D_2_EHPA have a greater impact on the membrane process than the concentration of TBP. Moreover, other parameters of the membrane process, e.g., changes in the acidity of the stripping phase, can significantly affect the efficiency of the process.

**Figure 1:**
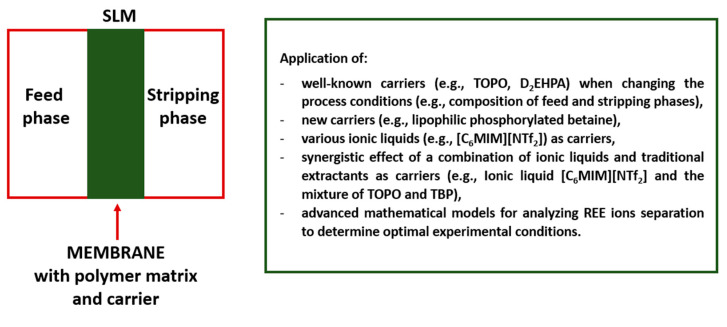
Main modifications related to the formation of SLMs intended for REEs recovery (since 2019, prepared based on the references contained in this section).

Since the membrane processes conducted using various SLMs are affected by many factors (e.g., membrane composition, form and properties, feed phase properties—e.g., whether the separation concerns a single REE or a mixture of REEs from model single or binary solutions, or from real multi-component solutions, stripping phase composition and pH, etc.) studies that support experimental work with the development of advanced mathematical models for analyzing metal ion separation have also been recently performed [23,49]. The application of mathematical models may be particularly useful when “designing” membranes that could potentially be implemented in the future on an industrial scale. In the case of SLM-based methods, in addition to the efficiency of the process, the stability of the developed membrane also plays an important role because the need to frequently replace membranes will increase the costs of REE ion recovery and may also constitute a technical problem. The stability of SLMs during long operation may be affected by various parameters, e.g., the nature of support and organic phase of the membrane, and conditions in which the membrane operates. Therefore, choosing the right separation method and optimal experimental conditions can be a challenge and, as noted by Kostanyan et al. [20], “to select an appropriate method for solving the set separation problem, as well as for its optimal design, preliminary mathematical modeling is necessary”. Recently, Tyagi et al. [50] used an artificial neural network coupled with a genetic algorithm for modeling and optimization of the separation of neodymium ions with the use of a supported liquid membrane (with TOPO carrier) and, after the analysis of the effects of different input factors on the transport rate, determined the optimum set of parameters that provided the maximum extraction. Figure 1 shows the main modifications related to the formation of SLMs intended for REEs recovery (since 2019).

The achievements related to the application of SLMs for the recovery of REEs described in this section indicate that further research on the possibility of using the synergistic effect of various carriers (including ILs) and the utilization of advanced mathematical modeling methods to determine the optimal conditions for conducting membrane processes may be of key importance for the development of these eco-friendly separation methods.

## 3. Utilization of Emulsion Liquid Membranes for the Recovery of REEs

In the case of separation processes carried out using ELMs, the liquid membrane phase, which contains carriers, is dispersed by forming an emulsion. In general, in ELMs, the aqueous stripping phase (internal phase) is encapsulated (as microdroplets) in large droplets of a liquid membrane phase moving in the aqueous feed phase (external phase, containing an analyte, e.g., REE ions). However, systems are also known in which the feed phase is encapsulated (as microdroplets) in large droplets of the liquid membrane phase moving in a continuous stripping phase [20]. ELMs are double emulsions as they constitute either a water-in-oil-in-water (w/o/w) system or an oil-in-water-in-oil (o/w/o) system. In the case of a water-in-oil-in-water system, in the first stage, the internal aqueous stripping phase and the membrane phase form water-in-oil emulsion globules, and then dispersing them in the external continuous feed phase leads to the formation of a w/o/w system. Both systems (w/o/w, o/w/o) are characterized by large contact areas [20,51,52]. In the ELM-based process, first, the separated substance (e.g., REE ions) migrates from the aqueous feed phase to the interface of the membrane on the feed phase side and then reacts with the carrier molecules present in the membrane phase and forms complexes at the interface. In a further stage, the complexes are transferred through the entire membrane and, after reaching the interface of the membrane on the stripping phase side, they dissociate, releasing the analyte (to the stripping phase) and “regenerating” the carrier. The efficiency of ELM processes depends on many factors, such as, for example, carrier type and concentration, the ratio of internal/external phase volume to the membrane phase volume, the composition and concentration of the internal phase, or the membrane process time. The concentration of surfactants, which are often added to the membrane phase to improve the stability of the emulsion (stabilization as a result of micellization) is also an important parameter [51,52,53]. Although emulsion liquid membrane processes depend on many factors and, consequently, their optimization may be fairly time-consuming, ELMs have many advantages, the most important of which include integration of extraction and stripping processes, usually high extraction efficiency, high surface area, relatively low consumption of chemicals, no need to use advanced devices, and low energy consumption [21,51]. Limitations to the use of ELMs, especially on a larger scale, are usually related to the stabilization of the fragile emulsion, loss of carriers, problems related to de-emulsification, or the recovery of solvents for reuse [21]. Due to the advantages of ELMs, the relatively few disadvantages, and the possibility of easy modifications of the separation system to adapt it to the properties of the source from which ions are recovered (e.g., the possibility of using various carriers), techniques based on emulsion liquid membranes to recover REE ions from different solutions have been intensively used. The research concerned, inter alia, the possibility of recovering REEs from different sources (leachates of ores, wastes, ashes, or model solutions) using various carriers. For example, Smith et al. [54] performed experiments on recovering REEs from leachates of coal fly ash with the use of D_2_EHPA (dissolved in kerosene or mineral oil) in three different separation processes, i.e., in standard solvent extraction and two membrane processes based on the use of an emulsion liquid membrane and a supported liquid membrane. In the case of ELM, the said authors used the hydrophobic surfactant Span 80 to stabilize the emulsion and mineral oil instead of kerosene. ELM separation of REEs performed by immersing an acid-in-oil emulsion in the leachate led to obtaining similar recovery percentages of ions of individual rare earth elements as in solvent extraction experiments. Recovery values using ELM varied for different REEs, ranging from a few percent (e.g., Lu) to almost 100% (e.g., Eu) after 60 min of running the process. Additionally, the recovery of REEs was faster in ELM separation compared with the SLM process. However, the obtained results also indicated that ELM was more selective for light REEs, whereas SLM was more selective for heavy REEs. Based on a flux-based model, the authors reported that recovery rates were limited by the affinity of the ions for the carrier in the case of the SLM process, and by diffusive mass transfer in ELM separation. Moreover, although both ELM and SLM enabled the effective separation of REEs from leachate containing other competing metal ions, an emulsion liquid membrane could be implemented with mineral oil, a solvent that is less hazardous than kerosene, which makes this method more eco-friendly. The development of efficient and environmentally safe methods that allow the recovery of REEs from an unconventional source, such as fly ash, is important because the increasing amount of fly ash produced around the world entails the need for its recycling and disposal, and recovering rare earth elements from this type of resource could be a way to secure supplies of these necessary row materials [55]. The study investigated the possibility of replacing traditional organic solvents (e.g., kerosene, toluene, hexane, etc.) used in the formulation of ELMs with more environmentally safe solvents that are non-toxic, non-volatile, reusable, and degradable in nature (e.g., vegetable oils), which is one of the currently observed trends. The concept of green emulsion liquid membranes (GELMs) was even introduced in relation to techniques based on the use of ELMs and green solvents, and these techniques are a promising solution for the removal of various contaminants (including metal ions) from aquatic streams [56]. Karmakar et al. [52] investigated the possibility of using various oils (i.e., mustard oil, coconut oil, and palm oil) as the organic phase and two dyes, such as aniline yellow (AY) and benzene azo naphthylamine (BAN), as carriers in ELMs intended for the separation of Dy(III) ions from other lanthanoid ions such as La(III), Ce(III), Ce(IV), Pr(III), Sm(III), and Gd(III) present in model aqueous solutions. They conducted a two-stage analysis, wherein the first stage was related to the determination of the ability of dyes having free amino groups to bind ions of individual REEs (UV-Vis spectrophotometry analysis). The experiments were performed at different pH (pH = 1, 3, or 5) conditions, and the results indicated that complexation occurred at pH = 3 and pH = 5 only for Pr(III) and Dy(III) ions with both dyes. In the second stage, ELMs that, in addition to the appropriate oil and carrier, contained a non-ionic surfactant, i.e., Triton X-100, stabilizing the emulsion were used in the separation processes. It was found that extraction efficiency depended on the selection of both the oil and carrier, as well as on stirring time, the pH of the medium, and the concentrations of the feed solution. The best results for selective extraction of Dy ions were obtained for an ELM containing mustard oil and AY dye at pH = 3 (extraction efficiency of 90%). Additionally, after the ELM process, the oil-rich phase was demulsified using a mixture of organic solvents, and the extracted metal ions were back-extracted (with cation exchange resin) with an efficiency of 88%. Raji et al. [57] reported the possibility of using sunflower oil as an environmentally friendly and sustainable solvent for the pertraction of neodymium (Nd) from an aqueous solution through an ELM containing a mixture of mono-(2-ethylhexyl) ester of phosphoric acid (M_2_EHPA) and D_2_EHPA as the carrier and Span 80 as the surfactant. Their results demonstrated that under optimal experimental conditions, almost all of Nd present in the external aqueous phase was extracted in less than 20 min of the process. Subsequent modifications of ELMs involve the utilization of other carriers. For example, Laguel and Samar [58] applied an ELM with Cyanex 302 as the carrier, Span 80 as the surfactant, kerosene as the diluent, and sulfuric acid solution as the internal aqueous phase for the removal of europium(III) from aqueous solutions. They focused on the optimization of various parameters influencing ELM formation and stability for increasing the removal of Eu(III) and reported that in optimal conditions (surfactant concentration of 3%, carrier concentration of 0.3%, internal phase composition of 0.5 N H_2_SO_4_, stirring speed of 200 rpm), the extraction efficiency was about 92%.

**Figure 2:**
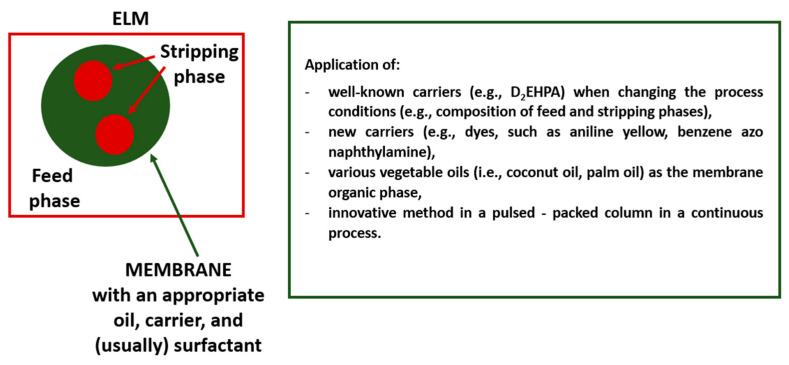
Main modifications related to the formation and use of ELMs intended for REEs recovery (since 2019, prepared based on the references contained in this section).

In addition to modifying ELM separation processes by changing the composition of membranes (replacing some components with others, e.g., exchanging organic solvents and commonly utilized carriers with vegetable oils and ILs, respectively) and conditions of experiments, research is also carried out to intensify the processes in which membranes containing well-known carriers and solvents are used. For example, Sadehlari et al. [59] developed a novel method for an emulsion liquid membrane in a pulsed-packed column in a continuous process, which was used for dysprosium (III) extraction. The ELM applied in the experiments consisted of common components such as D_2_EHPA as the carrier, Span 80 as the surfactant, kerosene as the diluent, and nitric acid as the internal phase. In the studies, flooding conditions and holdup at flooding points of the packed column were characterized, as well as operational parameters affecting the flooding points, such as pulsation intensity and the flow rate of the continuous and dispersed phases. The obtained results showed that under optimal process conditions, as much as 99.7% of dysprosium was extracted, and what is more, the extraction efficiency of the recovered membrane was as high as the unused membrane phase. The results are promising and indicate new possibilities for using ELMs on a larger scale in the future. Moreover, once the parameters influencing the novel membrane process have been determined, the new method may also potentially be used for efficient and continuous extraction of other REEs. Figure 2 shows the main modifications related to the formation and use of ELMs intended for REEs recovery (since 2019).

It can be assumed that further research on the possibility of using novel non-toxic chemical compounds as carriers and replacing traditional organic solvents with environmentally safe vegetable oils will be one of the future trends in the modification of ELMs intended for the recovery of REEs.

## 4. Polymer Inclusion Membranes in the Recovery of REEs

Polymer inclusion membranes are a type of LM in which the liquid phase (usually containing, in addition to the carrier, a plasticizer) is held within the polymeric network of a polymer matrix. PIM-based separation methods are attracting interest among researchers and are relatively widely used, both for the recovery of valuable materials (e.g., precious metals) and for the removal of dangerous contaminants (e.g., pharmaceuticals) from various aqueous solutions (e.g., leachates of various types of waste and wastewater), due to their numerous advantages, such as the possibility of carrying out simultaneous extraction and back extraction, high efficiency of separation processes, stability of membranes, the possibility of their repeated use after regeneration, and relatively low costs related to the preparation and operation of membranes. Moreover, they require the use of only small amounts of organic solvents and can be relatively easily modified by replacing the components with more environmentally safe substitutes, which makes their use fit into the so-called “green chemistry” [22,60,61,62,63]. As noted by Kujawa et al. [64], “PIMs constitute the most promising candidates for sustainable systems dedicated for the REE separation”. One of the types of modifications of PIMs whose effects can be considered eco-friendly is the use of ionic liquids instead of traditional carriers. Furthermore, in the case of some PIMs, the utilization of ILs also allows the membrane to be given appropriate plastic properties, so there is no need to use an additional plasticizer, which is beneficial from the point of view of both environmental protection and economics [22,64]. The PIM-IL system intended for the separation of REEs ions was, inter alia, used by Wang et al. [65], who applied ionic liquid Cyphos IL 104 (trihexyl (tetradecyl) phosphine bis (2,4,4-trimethyl-amyl)–phosphonate) as the carrier in a polymer inclusion membrane based on a PVDF matrix for adsorption and separation of heavy rare earth elements such as lutetium (III) and ytterbium (III). They reported that the addition of Cyphos IL 104 promoted the hydrophilicity of the membrane (observed reduction in the water contact angle). The surface of the formulated PIMs exhibited improved antifouling properties and good reusability, and under optimal experimental conditions, the developed method (Cyphos IL 104/PVDF PIM) enabled the effective separation of REE ions. Makówka and Pośpiech [66] used Cyphos IL 104 in PIM with a CTA polymer matrix for the separation of Ce(III) from a model solution containing La(III), Cu(II), Co(II), and Ni(II). They found that the PIM with optimal composition (20.0 wt% CTA, 55.0 wt% NPOE, and 25.0 wt% Cyphos IL 104) enabled the separation of Ce(III) (transport efficiency of 67%), and the separation followed the order: S_Ce/La_ < S_Ce/Cu_ < S_Ce/Co_ < S_Ce/Ni_. Modifications of the composition of PIMs may also involve polymers, and such a change should result in improved membrane properties affecting the efficiency of the membrane process. For example, Chen et al. [67] synthesized a novel PIM incorporating the hydrophilic additive random copolymer poly(vinyl alcohol-co-ethylene) (EVOH) with Cyanex 272 carrier and applied it for the separation of ytterbium and lutetium ions. They found that an appropriate amount of EVOH contributed to larger surface pores and internal channels of the membrane. However, despite the improvement in some important properties of the formulated EVOH-containing membrane, carrier leakage was also observed, which led the aforementioned authors to the conclusion that the Cyanex 272 carrier used may not be the best choice in terms of PIM stability.

Another solution that has been intensively investigated in recent years in membrane technologies is the replacement of organic solvents used in the formulation of membranes with more ecological “greener solvents”. In general, solvents play an important role in dissolving membrane components and affect the final properties of the membrane (e.g., pore size and porosity), consequently influencing the membrane process. The group of green solvents includes ILs but also deep eutectic solvents (DESs), which are increasingly used due to their properties [68]. DESs, which are mixtures containing at least one hydrogen bond acceptor and one hydrogen bond donor, are characterized by, among others, low toxicity, high biodegradability, and simple preparation, with the possibility of using naturally occurring, relatively cheap chemical compounds [69]. Chen et al. [70] recently reported that DESs can also be used differently in the case of PIMs, contributing to the improvement in membrane properties. In the performed experiments, they used a natural deep eutectic solvent (NADES, from betaine and lactic acid) to regulate and control the coagulation bath of a PIM (PVDF/Cyanex 272 membrane, formulated using the non-solvent-induced phase separation method) intended for the separation of Nd, Sm, and Dy ions. The obtained results showed that the use of the stage of coagulation bath with NADES significantly improved the properties of the membrane (pores on the surface were richer and larger, and the surface layer was more hydrophilic). Consequently, the utilization of a small amount of NADES (optimally 5%) in the coagulation bath allowed the formation of a more porous PIM with higher surface hydrophilicity, optimized permeability, and high separation efficiency toward REEs.

Research is also being carried out into the possibility of using other carriers in PIMs intended for the recovery of various REEs. For example, Huang et al. [71] examined di(2-ethylhexyl) phosphinic acid as a novel carrier, acting also as a plasticizer in a PIM-containing poly(vinylidene fluoride) (PVDF) matrix designed for the transport of Lu(III). The research concerned determining the optimal composition of the PIM (the ratio of the amount of polymer to the amount of carrier), the optimal experimental parameters of the Lu(III) transport process, and the stability and reusability of a polymer inclusion membrane with an optimal composition (cyclic extraction-stripping experiments). Additionally, to enhance the transport efficiency of Lu(III), a dual-membrane transport apparatus was designed and utilized. The results of scanning electron microscopy showed that the membrane with 40 wt% of the carrier (and plasticizer) had a hierarchically ordered porous structure on its glass-side surface. Such membrane surface morphology increased the contact surface of PIM with the solution, which had a positive effect on the efficiency of the process. Interestingly, Lu(III) transport efficiency was different in the opposite transport direction. The obtained results indicated that the transport efficiency of Lu(III) depended on many factors, such as, inter alia, the composition of PIM and the morphology of the membrane surface, transmission direction, and the use of a dual-membrane transport apparatus, which allowed for a significant reduction in the duration of the process (about 85% of Lu(III) was recovered after 5 h of the process without the apparatus and after 3 h of the process with the application of the apparatus). The possibility of shortening the time of the membrane process may be of significant economic importance, as may the use of a carrier that also serves as a plasticizer, especially in the case of processes carried out on a larger scale. Huang et al. [72] also used the same composition of PIMs (40 wt% di(2-ethylhexyl) phosphinic acid, 60 wt% poly(vinylidene fluoride)) for selective transport of Lu(III) from the model acidic feed solution containing similar concentration of La(III) and Sm(III). They found that although the formulated PIM enabled efficient recovery of Lu(III) (91% after 36h of membrane process), a small amount of Sm(III) (about 5%) was also transported through the membrane. Additionally, they developed a relatively simple method for regenerating a used membrane and demonstrated (based on SEM) that the regenerated PIM had similar properties to the membrane before its use in the transport process. Developing a method that enables the effective separation of specific ions from a feed solution containing other rare earth elements is an important achievement because both primary and secondary sources of REEs usually contain many components. Making it possible to effectively regenerate a membrane without deteriorating its separation properties is also an important milestone.

**Figure 3:**
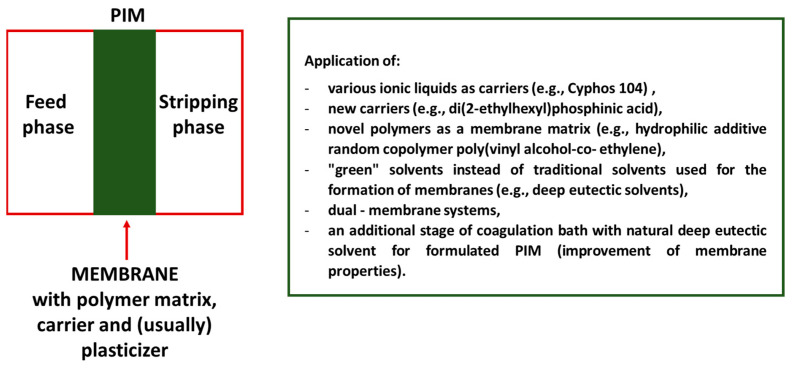
Main modifications related to the formation and use of PIMs intended for REEs recovery (since 2019, prepared based on the references contained in this section).

However, since systematic efforts are being taken to improve the effectiveness of membrane processes, reduce the amount of chemical reagents used, and shorten the duration of the process, other solutions have also been introduced. For example, Croft et al. [73] developed micro polymer inclusion beads (µPIBs), which are similar in composition to PIMs but are characterized by higher specific surface area and, consequently, enable faster extraction and back-extraction. µPIBs containing well-known extractant D_2_EHPA (30–80 wt%) and poly(vinyl chloride) (PVC) or poly(vinylidene fluoride-co-hexafluoropropylene) (PVDF-HFP) (70–20 wt%) as the polymer matrix were formulated with the application of the phase inversion microfluidic method and used for La(III) ion extraction. A comparison of results of selective extraction of La(III) ions performed in the same experimental conditions, obtained with the application of µPIBs with 45 wt% of D_2_EHPA and 55 wt% of PVC, and the utilization of identical composition, traditional PIM having the same mass, showed that micro polymer inclusion beads exhibited a 6 times higher surface area and a 4.4 times higher initial mass transfer rate. However, in the case of µPIBs, additional acidification of the NaCl desolvation and delivery solutions was necessary to prevent D_2_EHPA carrier leaching. In subsequent research, Croft et al. [74], while characterizing micro polymer inclusion beads using thermogravimetric analysis, discovered, inter alia, that the presence of Na^+^ in both PVC and PVDF-HFP based μPIBs negatively influenced the extraction efficiency of La(III) ions. In that study, they also found that this adverse process can be prevented, e.g., by washing the μPIBs with 1 M sulfuric acid after their formulation to remove the undesirable Na^+^ ions. Therefore, the membrane processes are influenced by many factors, inter alia, the form of the membrane, and the change in this form causes other factors (e.g., the content of Na^+^ ions) to play an important role. When developing a new method for the recovery of REEs from a variety of sources, all these factors must be taken into account, which means that the preparatory procedure is usually laborious and time-consuming, although the method resulting from the research may be relatively simple. Figure 3 shows the main modifications related to the formation and use of PIMs intended for REEs recovery (since 2019).

Summarizing, separation processes based on the application of PIMs are becoming more and more popular, and the possibility of relatively easy modifications to membranes (e.g., the use of various compatible carriers and polymers, the use of ILs for improving membrane stability, etc.) and experimental conditions (e.g., stripping phase composition and pH) allows their effective use for the removal/recovery of various substances from aqueous solutions (e.g., lithium and magnesium ions, heavy metal ions, REEs, etc.) [22,75,76]. However, it should be emphasized that various membrane techniques are being systematically developed, and new solutions are being introduced. For example, for water purification, the possibility of using more advanced materials (e.g., inorganic–organic hybrid membranes, and MXene-based hybrid nanomaterials) has been recently investigated [77,78].

## 5. Conclusions

Although various types of liquid membranes have been used in recent years for the recovery of rare earth elements from aqueous solutions, research is systematically carried out aimed at modifying LM-based processes to increase their efficiency, reduce the negative impact on the environment, and minimize the costs of membrane formulation and application. Many of the achievements regarding the effective separation of REEs with the application of LMs result from the possibility of using various chemical compounds as carriers (e.g., well-known extractants or novel substances) and the synergistic effect of a combination of two/three different carriers. The opportunity for introducing changes in relation to the polymers used and solvents utilized in the membrane formulation (e.g., replacing classic, usually toxic, organic solvents with so-called “green” solvents) was also investigated. One of the observed trends in relation to LMs intended for REEs recovery is the use of ionic liquids as effective and eco-friendly carriers (in SLMs, ELMs, and PIMs), which can also act as PIM plasticizers. The dual role of ILs in PIMs consequently reduces the costs of membrane formation. In addition to research on changing the LM components, attempts were also made to modify the membrane surface by, for example, using an additional step of coagulation bath with a natural deep eutectic solvent, which significantly improved the PIM properties and strongly influenced the efficiency of the membrane process. Other, recently introduced solutions that strongly influenced LM processes were the utilization of a dual-membrane transport apparatus (in the case of PIM) or the application of a pulsed-packed column (in the case of ELM). Because LM-based processes depend on many factors (the composition and form of the membranes, the properties of the recovered REEs, the composition and properties of the feed and stripping phases, the duration of the process, etc.), designing membranes intended for the recovery of specific rare earth elements from specific sources can be a time-consuming and labor-intensive process. Therefore, one of the observed tendencies when designing LMs is the use of a mathematical modeling methods, in which based on available data, the impact of individual factors on the REEs transport process can be analyzed, and then the set of optimal parameters with the maximum extraction efficiency can be determined.

Examples of the application of liquid membranes for the recovery of REE ions included in this work clearly demonstrate that these techniques, after establishing optimal conditions for conducting membrane processes, are efficient and, in many cases, enable highly selective separation of the desired components from complex, multi-component solutions. LM-based membrane processes are versatile and have the potential to be used on a larger scale in the future, e.g., industrially. Moreover, LM methods are considered eco-friendly due to, inter alia, the possibility of using “green” solvents during membrane formulation and the ability to reuse membranes after regeneration. However, due to a number of problems associated with the use of LMs (e.g., leaching of carriers, insufficient stability of membranes, changes in membrane properties after regeneration, reduced effectiveness of membranes in the presence of additional components, etc.), further research in this area is necessary.

## Figures and Tables

**Table 1:** Examples of various compounds, including ILs, used as carriers in SLMs intended for the recovery of different REEs.

Membrane Carriers	Removed Metal Ions	Main Findings	Reference
Ionic liquid [C_6_MIM][NTf_2_], D_2_EHPA, or TBP	Cerium ions	Applied IL improves the extraction procedure and increases the permeation coefficient through the SLM. It selectively facilitates the transport of Ce(III) ions through the SLM.	[43]
Ionic liquid[C_6_MIM][NTf_2_] and the mixture of TOPO and TBP	Neodymium and Praseodymium ions	The highest permeability coefficients were obtained with the synergistic system containing [C_6_MIM][NTf_2_], TOPO, and TPB extractants. The formulated SLM was efficient and stable and could be used to recover REEs from the leaching solution of NdFeB permanent magnets.	[44]
		Extraction efficiency under optimal conditions was ~90%.	
Ionic liquid [C_6_MIM][NTf_2_] and D_2_EHPA and TBP	Gadolinium ions	Membrane efficiency increases with the synergistic effect of ionic liquid with organophosphorus extractants.	[45]
		Extraction efficiency under optimal conditions was ~73%.	
[C_6_MIM][NTf_2_], TBP, and D_2_EHPA	Yttrium ions	SLM membranes could be applied as a cost-effective and straightforward method for the extraction of yttrium ions from the leachate of fluorescent lamp waste.	[46]
Cyanex 923	Yttrium and Europium ions	The application of SLMs as an additional step prevented the loss of REEs during the leaching of YOX fluorescent lamp waste and improved the recovery of Eu(III) and Y(III) ions. Extraction efficiency under optimal conditions was ~96%.	[47]
Cyanex 272	Scandium ions	Scandium was separated from a multi-metal solution bearing copper, nickel, cobalt, zinc, iron, and manganese ions. Sc recovery using Cyanex 272 with HFSLM was 99.9%	[48]

Where: [C_6_MIM][NTf_2_]—1-hexyl-3-methylimidazolium bis[(trifluoromethyl)sulfonyl]imide, D_2_EHPA—di-(2-ethyl hexyl) phosphoric acid, TBP—tri-n-butyl phosphate, TOPO—tri-octyl phosphine oxide, Cyanex 923—a mixture of trialkyl phosphine oxides, Cyanex 272—bis (2,4,4-trimethylpentyl) phosphinic acid.

## Data Availability

Not applicable.
